# Modelling long-term cost-effectiveness of health promotion for community-dwelling older people

**DOI:** 10.1007/s10433-019-00505-1

**Published:** 2019-02-23

**Authors:** Magnus Zingmark, Fredrik Norström, Lars Lindholm, Synneve Dahlin-Ivanoff, Susanne Gustafsson

**Affiliations:** 1 Health and Social Care Administration, Municipality of Östersund, 83182 Östersund, Sweden; 2 Department of Public Health and Clinical Medicine, Epidemiology and Global Health, Umeå University, 90187 Umeå, Sweden; 3 Department of Health and Rehabilitation, Institute of Neuroscience and Physiology, The Sahlgrenska Academy at the University of Gothenburg, Gothenburg, Sweden; 4 University of Gothenburg Centre for Ageing and Health (AgeCap), Gothenburg, Sweden

**Keywords:** Senior meetings, Preventive home visit, QALYs, Booster session, Health economy, Multi-professional

## Abstract

The effectiveness of health promotion for community-dwelling older people is well documented; however, there is a general lack of health economic evaluations. The aim of the present study was to evaluate long-term cost-effectiveness over 4 years of two health promoting interventions: senior meetings and a preventive home visit, for community-dwelling older people in relation to no intervention. We applied a Markov model including five states defined in relation to level of dependency of home help and place of residency. The model included transitions between dependency states, scores for quality of life and societal costs for each state, intervention costs and intervention effects for two formats of health promoting interventions. For each intervention and a no-intervention control group, we calculated the accumulated quality-adjusted life years (QALYs) and societal costs over 4 years. Sensitivity analyses included higher intervention costs, lower intervention effects and additional intervention costs and effects related to booster sessions. The results of all analyses indicated that health promotion implemented for community-dwelling older people in the format of senior meetings or a preventive home visit was cost-effective. Both interventions lead to QALY gains and reduce societal costs at any follow-up over 4 years, and thus, resources can be used to implement other interventions. The most important factor for the magnitude of QALY gains and cost savings was the intervention effect. Yearly booster sessions implemented for those persons who maintained their level of functioning extended the intervention effects adding additional QALYs and further reducing societal costs.

## Introduction

Health promotion for community-dwelling older people has the potential to positively affect various facets of functioning, health and independence (Beswick et al. 2008). While sharing a general focus on the promotion of active and healthy ageing, health promoting interventions have varied with regard to their specific content, duration and mode of delivery. For example, in a recent trial, three formats of health promoting occupational therapy (i.e. discussion group, activity group and individual intervention) were found to result in better effects than no intervention (Zingmark et al. 2014). No format was identified as superior regarding effects, but a one-session discussion group was identified as the most cost-effective intervention (Zingmark et al. 2016a, b). The trial “Elderly in the risk zone” provides another example in which both senior meetings and a preventive home visit, delivered in a multi-professional context, were found to result in better effects than no intervention (Gustafsson et al. 2012). Given the results of recent trials (Gustafsson et al. 2012, 2013; Zingmark et al. 2014), it seems as if health promotion for community-dwelling older people may include only a few sessions and still be effective. Thereby, interventions may be implemented at a relatively low cost, especially since a group format seems to result in positive effects on a broader range of outcomes than an individual format (Gustafsson et al. 2012; Zingmark et al. 2014). Since a persons’ functional level is associated with both health-related quality of life (HRQoL) and societal costs (Lindholm et al. 2013), it is also important to consider the cost-effectiveness of interventions.

Decisions on which interventions to implement can be guided by the use of health economic evaluation methods, i.e. when both health effects and costs are considered (Drummond et al. 2005). Data for a health economic evaluation can be included as part of the data collection in a clinical trial (Drummond et al. 2005). However, due to practical reasons (e.g. attrition, costs, logistical issues), a single trial can seldom provide all evidence needed to evaluate long-term cost-effectiveness (Briggs et al. 2006). Without a sufficiently long follow-up period, there is a risk that only the full cost for implementing the intervention is included, whereas the effects and societal costs over the long term are excluded (e.g. Flood et al. 2005). To overcome practical and ethical concerns related to a trial that is extended over many years, an alternative approach is to implement decision modelling (Briggs et al. 2006). Decision modelling provides a framework for synthesizing already existing data (model parameters) gathered from various sources, e.g. probabilities for transitions between health states in a specific population (longitudinal cohort studies) and costs and HRQoL for each health state, intervention effects, e.g. in terms of how probabilities for changes in health are affected. By synthesizing model parameters, it is possible to calculate the consequences (e.g. costs end effects) of the options under study (e.g. intervention vs. no intervention) (Johnell et al. 2003).

While the effectiveness of health promotion for community-dwelling older people is supported by several trials, there is a general lack of health economic evaluations (Dubas-Jakóbczyk et al. 2017). The existing evidence indicates that health promotion for community-dwelling older people is cost-effective in the short term (Hay et al. 2002; Zingmark et al. 2016a, b). However, given the potential long-term benefits of health promotion there is an urgent need to explore cost-effectiveness over the long term to guide decision making.

The aim of the present study was to evaluate long-term cost-effectiveness over 4 years of two health promoting interventions, senior meetings and a preventive home visit, for community-dwelling older people compared to no intervention.

## Methods

To evaluate cost-effectiveness of health promoting interventions implemented for community-dwelling older people compared to no intervention, we applied a Markov model previously used in another modelling study (Zingmark et al. 2016b). The development and full details of the model are described elsewhere (Zingmark et al. 2016b). The model parameters for this study included probabilities for transitions between dependency states (Raîche et al. 2012) (Table 1), scores on HRQoL (Andersen et al. 2004; Honkanen et al. 2006; Szanton et al. 2011; Fusco et al. 2012; Zingmark et al. 2014), societal costs for each state (Lindholm et al. 2013) (Table 2), intervention cost and intervention effects. The intervention effect was based on a randomized controlled trial (Dahlin-Ivanoff et al. 2010).

**Table 1:** Transition probabilities^a^ for annual transitions between states of dependency

	Mild dependency	Moderate dependency	Severe dependency	Total dependency	Dead
Mild dependency	0.79	0.13	0.03	0.02	0.03
Moderate dependency	0.08	0.82	0.03	0.01	0.06
Severe dependency	0.02	0.12	0.61	0.11	0.14
Total dependency	0.00	0.03	0.18	0.63	0.16
Dead					1.00

^a^The transition probability is the probability for a person, over a 1-year period, to transition between states. For example, a person in the mild dependency state has a probability of 0.79 to remain in the mild dependency state and a probability of 0.13 to transition to the moderate dependency state from 1 year to the following year

**Table 2:** Estimates of total costs (€) for 1 year including informal care, health care, home care and special accommodation (Lindholm et al. 2013), and HRQoL^a^ scores for each state in the Markov model

Markov state	Total costs (€)	HRQoL scores	References HRQoL scores
Mild dependency	2600	0.77	Zingmark et al. (2014)
Moderate dependency	7801	0.60	Fusco et al. (2012) and Szanton et al. (2011)
Severe dependency	20,708	0.47	Andersen et al. (2004)
Total dependency	62,407	0.41	Andersen et al. (2004) and Honkanen et al. (2006)
Dead	0	0.00	

^a^Health-related quality of life

In health economic evaluation, quality-adjusted life years (QALYs) is a commonly used outcome that was developed as a combined measure of HRQoL and time (Weinstein and Stason 1977). Based on model parameters, the accumulation of QALYs and costs was modelled over 4 years from a societal perspective in hypothetical cohorts of community-dwelling older people who received no intervention or a health promoting intervention in the format of either senior meetings or a preventive home visit. The design and reporting of the trial was made in accordance with the Consolidated Health Economic Evaluation Reporting Standards (CHEERS) statement (Husereau et al. 2013).

### Model structure

**Fig. 1:**
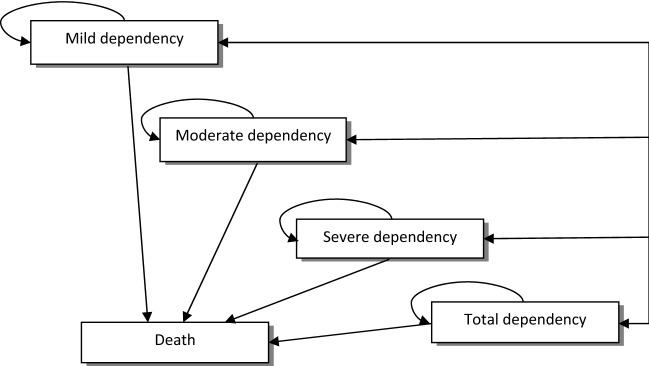
Markov model of transitions between states of dependency and death. Each arrow represents a possible transition (i.e. recovery, stability or decline) between two states over a 1-year cycle. *Mild dependency* refers to a state in which a person is independent in basic activities of daily living (BADLs), is dependent in no more than a single instrumental activity of daily living (IADL) and needs help no more than one time per week. *Moderate dependency* refers to a state in which a person is independent in BADLs, is regularly dependent in more than one IADL and needs help more than one time per week. S*evere dependency* refers to a state in which a person is dependent in one BADL and more than one IADL and needs help one or several times per day. *Total dependency* refers to a state in which a person is dependent in BADLs and IADLs and needs help one or several times per day and live at a special housing

The Markov model included five states which were defined according to level of dependency in basic activities of daily living (BADL) (e.g. bathing, dressing, toileting), instrumental activities of daily living (IADL) (e.g. cleaning, shopping, cooking) and place of residency because these aspects have been found to impact self-rated health as well as costs related to health and social care (Lindholm et al. 2013). The states modelled were: *mild dependency,* which refers to a state in which a person is independent in BADLs, is dependent in no more than a single IADL and needs help no more than one time per week. *Moderate dependency* which refers to a state, in which a person is independent in BADLs, is regularly dependent in more than one IADL and needs help more than one time per week. S*evere dependency* refers to a state in which a person is dependent in at least one BADL and more than one IADL and needs help one or several times per day. *Total dependency* refers to a state in which a person is dependent in BADLs and IADLs, needs extensive help throughout the day and lives in ordinary or special housing. The final state was *death*. Overall, the model illustrates a declining process towards increasing disability. However, the process towards increasing disability among older people involves both recovery and decline (Hardy and Gill 2004). Therefore, our model included transition probabilities for recovery, stability and decline over a 1-year period. Possible transitions are displayed in Fig. 1. All participants started in the mild dependency state. The cycle in the model was 1 year.

### Transition probabilities

The probabilities for yearly transitions were derived from a Canadian study by Raîche et al. (2012), including a cohort of 1410 persons 75 year or older from the general population who were identified as having a risk for functional decline, and were followed over 4 years. Based on a Canadian classification system for disability (Dubuc et al. 2006), annual transition probabilities for recovery, stability and decline were calculated in the cohort. Based on the originally reported transition probabilities (Raîche et al. 2012), we recalculated transition probabilities for our five-state Markov model (Table 1), also reported elsewhere (Zingmark et al. 2016b). The relation between each level in our model and the Canadian classification system was as follows: mild dependency included profile 1 (independence in ADL, difficulties with IADL), moderate dependency included profiles 2–5 (ranging from supervision in IADL to dependency in IADL), severe dependency included profiles 6–9 (dependency in IADL and ADL limitations ranging from difficulties to a need for help), and total dependency included profiles 10–14 and long-term care facility (ranging from extensive need for help in ADL and dependency in IADL to total dependency).

### Health-related quality of life and societal costs

Both HRQoL and societal costs are related to level of dependency. Previous studies indicate that a decline in ADL (Fusco et al. 2012) and loss of independence (Andersen et al. 2004; Shearer et al. 2012) has a negative impact on HRQoL. Published data from the peer-reviewed literature was used to assign each state in the Markov model an estimated score for HRQoL on a scale ranging from 0 to 1 (Drummond et al. 2005) (Table 2). For the state *mild dependency*, we used baseline data (SF-12) from a trial including 177 community-dwelling older people (Zingmark et al. 2014). For the state *moderate dependency*, we approximated how a decline in ADL and IADL impacted HRQoL (EQ-5D) (Szanton et al. 2011; Fusco et al. 2012). For the states *severe dependency* and *total dependency*, we approximated HRQoL scores (EQ-5D) from data on decrements in HRQoL due to major loss of independence (Andersen et al. 2004) and move to a nursing home (Andersen et al. 2004; Honkanen et al. 2006) (Table 2). The HRQoL scores were multiplied by the time spent in each health state to derive a quality-adjusted life year (QALY) (Drummond et al. 2005).

Data from a Swedish cohort study were used to estimate costs for each state. As shown by Lindholm et al. (2013), function in terms of the level of dependency in ADL and IADL has a strong impact on total costs. Societal costs are given in Euro (€) and include direct costs for health care, home help, informal care and special accommodation (Table 2). The estimates of costs presented in Table 2 are based on the data presented in Figs. 1 and 3 by Lindholm et al. (2013). Indirect or intangible costs, e.g. the value of time for participating in the interventions, were not included. QALY scores and societal costs were discounted at 3% per year. The costs presented by Lindholm et al. were estimated in SEK based on the price level for 2008 and adjusted for inflation until July 2018. Costs were converted to Euros (€) based on the currency August 24, 2018, 100 SEK = 9.46 (€).

### Interventions

#### Senior meetings and one follow-up home visit

This intervention comprised four weekly health promoting senior meetings with no more than six participants in each group. The main purpose was to focus on two different areas, (1) information and discussion about the ageing process and its consequences, and (2) providing tools and strategies for solving the various problems that may arise on the home environment. A follow-up home visit took place about 2–3 weeks after the senior meetings in which individual participants had the opportunity to discuss group topics in more depth. The senior meetings were led either by an occupational therapist, a registered nurse, a physiotherapist or a qualified social worker, who jointly planned and carried out the intervention. In addition, on each senior meeting, another professional participated by holding a 30-min lecture, focusing on his/her speciality. A booklet especially designed for the intervention, i.e. information referring to the topics discussed, was used as a basis for the senior meetings (Dahlin-Ivanoff 2009).

#### Preventive home visit

This intervention was in the form of one home visit made by either a registered nurse, a physiotherapist, a qualified social worker or an occupational therapist. The visit was guided by a protocol (Gustafsson et al. 2012). During this visit, the participants received verbal and written information and advice about what the municipality could provide in the form of local meeting places, activities run by local associations, help and support of various kinds offered either by volunteers or by professionals employed by the municipality, physical training for seniors, walking groups, etc. They were also informed about assistive devices and adaptation of housing. Fall risks were identified together with the person, and advice on how to prevent falls was included in the home visit. Information was given about who they could contact for different problems. No further assessments were implemented.

### Intervention effect

The effects of the interventions were based on data from a previous trial (Dahlin-Ivanoff et al. 2010) using the ADL staircase (Sonn and Hulter Asberg 1991) to evaluate performance in daily activities. At baseline, all participants in each group were in the mild dependency state. In Table 3, original data are presented showing group sizes and transition patterns over the first year from baseline for the control, senior meetings and preventive home visit groups, respectively. The intervention effects were calculated as the relative risks for progression from mild dependency to increased levels of dependency 1 year after baseline for no intervention compared to senior meetings and a preventive home visit, respectively. For example, the risk for a transition from mild to moderate dependency in each group was as follows: control group 19/114 = 0.17, senior meetings 11/171 = 0.06 and preventive home visit 19/174 = 0.11. The relative risk for a transition from mild to moderate dependency was 0.35 (0.06/0.17 = 0.35) for participants in senior meetings relative to the control group and 0.65 (0.11/0.17 = 0.65) for participants in a preventive home visit relative to the control group.

**Table 3:** Data on which the intervention effect was calculated including group size and the number of participants in each state 1 year after baseline

Intervention	Mild dependency, *n*	Moderate dependency, *n*	Severe dependency, *n*	Total dependency, *n*	Dead, *n*
Control, n = 114	87	19	6	0	2
SM^a^, *n* = 171	149	11	3	3	5
PV^b^, *n* = 174	150	19	1	0	4

^a^Senior meeting, ^b^preventive home visit

### Intervention cost

All intervention costs were adjusted for inflation and converted to Euros (€) based on the same procedure as described for societal costs (see above) (Table 4). The cost for salaries, 26.8 €/h, was based on an average of the gross mean income in Sweden for each of the involved professionals, i.e. a registered nurse, a physiotherapist, a qualified social worker and an occupational therapist. The time for preparatory education of interveners was 20 h per professional, in all 80 h. For home visits in both groups, costs were estimated for transportation, on average 6 km per home visit at a cost of 1.4 € per home visit.

**Table 4:** Items for estimates of total intervention costs (€) for senior meetings (SM) and a preventive home visit (PV)

Cost items	SM^a^ (*n *= 171)	PV (*n *= 174)
Education	2143	2143
Salaries^b^	21,071	9322
Travel costs interveners	234	238
Rented rooms	3222	0
Materials	623	634
Total intervention cost	27,293	12,337

^a^Senior meetings included five participants on average/meeting

^b^Include time for planning and the intervention

The approximated time for each senior meeting including preparations was 3 h. In addition, another professional participated by holding a 30-min lecture, focusing on his/her speciality on each senior meeting. It was approximated that each senior meeting was attended by a group of five participants. Thus, the total time for the four senior meetings was 14 h, equal to 2.8 h per participant. In all, there were a total of 34 groups and 136 senior meetings. For the follow-up home visit, we approximated that the total time including travel and preparations was 1 h 45 min per home visit. In all, the average time for the intervention in the senior meeting group including follow-up was 4.6 h per participant. For the senior meetings, the cost for rented rooms was estimated to be 24 € per session based on a recent trial (Zingmark et al. 2016a). The cost for the booklet used during the senior meetings was estimated to be 4 €. The time for the senior meetings was in the middle of the day during which public transport was free for seniors. Subsequently, there were no costs for transportation for participants.

For the home visits, we approximated that the total time including travel and preparations was 2 h per participant. The estimated cost for printed materials was 4 €.

Given the total cost for each type of intervention, the cost per participant was 160 € for senior meetings and 71 € for a preventive home visit. The cost for booster sessions was 19 € for one additional senior meeting and 71 € for one additional preventive home visit.

### Statistical analysis

The analysis was based on hypothetical cohorts in which all participants were in the state mild dependency at the beginning of the trial. We applied Microsoft^®^ Excel software (Menn and Holle 2009) to analyse the Markov model. In all, the model included the parameters presented above, i.e. transition probabilities, health-related quality of life, societal costs, intervention effects and intervention costs. In the main analysis, we estimated that the interventions yielded a one-time effect during the first year, resulting in a reduced risk for negative transitions. After the first year, all transitions followed the same pattern, i.e. the transition probabilities in Table 1, in both groups. For each intervention and compared to the no-intervention control group, we calculated the accumulated QALYs and societal costs over 4 years.

#### Sensitivity analysis

To acknowledge uncertainty in parameter estimates, we conducted three different types of sensitivity analyses. We hypothesized that a reduced intervention effect and an increased intervention cost would reflect real-world variation that could affect cost-effectiveness. For example, a consequence of dropouts leading to smaller group sessions than planned would have an impact on both the average cost for the intervention and also on how many persons would benefit from the intervention. For the sensitivity analysis, we assumed a 20% higher intervention cost and a 20% reduced intervention effect. We performed the analysis for each of the assumptions separately. In addition, we included an analysis of booster sessions since it has been suggested that booster sessions could be one strategy to maintain a higher level of intervention effect over time (Gustafsson et al. 2012). Therefore, we also conducted an analysis based on the assumption that booster sessions, i.e. one additional senior meeting or one additional preventive home visit, were implemented once a year for those who remained in the mild dependency state for years 2, 3 and 4, respectively.

For the analysis of booster session, we hypothesized the same additional intervention cost and intervention effect for years 2, 3 and 4 as for year 1.

## Results

The results of the main analysis, the sensitivity analyses and the analysis of booster sessions are presented in Table 5. In Table 6, the results are extrapolated to hypothetical cohorts of 100 people in each group. In addition to the reduced risk for a transition from mild to moderate dependency after 1 year, a slightly larger proportion of participants in any of the interventions remained in more favourable states during subsequent years (Table 6). In all, this effect led to a positive accumulation of QALYs and reduced societal costs from over the whole follow-up period for both interventions. Compared to no intervention, the senior meetings resulted in 0.054 QALYs gained over 4 years and lower societal costs amounting to 2283 € (approximately 230,000 € when extrapolated). The preventive home visit resulted in 0.048 QALYs gained over the same time period and lower societal costs amounting to 2091 € (approximately 210,000 € when extrapolated).

**Table 5:** Accumulated quality-adjusted life years (QALYs) and costs over 4 years for one person

Analysis	No intervention		SM		PV		SM		PV	
	QALYs^a^	Costs (€)	QALYs	Costs (€)	QALYs	Costs (€)	Incremental QALYs^b^	Incremental costs (€)^b^	Incremental QALYs^b^	Incremental costs (€)^b^
Main analysis	2.355	24,291	2.409	22,008	2.403	22,200	0.054	− 2283	0.048	− 2091
Sensitivity analyses										
Reduced intervention effect	2.355	24,291	2.396	22,600	2.389	22,847	0.041	− 1691	0.033	− 1444
Increased intervention cost	2.355	24,291	2.409	22,039	2.403	22,214	0.054	− 2251	0.048	− 2077
Booster session^c^	2.355	24,291	2.479	18,146	2.464	19,046	0.123	− 6145	0.108	− 5245

Incremental QALYs and costs are given for senior meetings (SM) and preventive home visits (PV) in relation to no intervention

^a^Quality-adjusted life years

^b^Incremental QALYs and costs were calculated for SM and a PV, respectively, in relation to no intervention

^c^Include an additional group session for SM and an additional home visit for PV for those who remained in the state mild dependency at years 1, 2 and 3, respectively

**Table 6:** Number of persons^a^ that remained in the mild dependency state or had transitioned to moderate dependency during years 1–4

Year	Mild dependency			Moderate dependency		
	Controls	SM	PV	Controls	SM	PV
1	79	87	86	13	7	8
2	64	69	69	21	17	18
3	52	56	56	26	24	24
4	43	47	46	29	28	28

^a^Based on hypothetical cohorts of 100 people in each group

When results were extrapolated, an additional three to five persons remain in the mild dependency state, for any of the interventions when compared to no intervention for the years 2–4 (Table 6).

During years 2–4, more persons in both intervention groups transition to moderate dependency compared to the control. However, when the states mild and moderate dependency were considered together, the number of persons in any of the intervention groups was higher than in the control group.

While both interventions reduced the risk for transitions from mild to moderate dependency during the first year, the results also indicated a small long-term effect on transitions to more severe health states. Over the whole period of 4 years, transitions to severe dependency, total dependency and death were more frequent in the control group (*n *= 28) than they were in either of the intervention groups (*n *= 25), (not presented in Table 6).

### Sensitivity analysis

The sensitivity analysis indicated that a higher intervention cost, for example due to a smaller group size than 5 persons, had a small impact on long-term costs and overall cost-effectiveness. The difference in cost per person over 4 years was 32 € for the senior meetings and 14 € for the preventive home visit. In contrast, the size of the intervention effect was a more critical parameter in terms of both fewer QALYs gained and reduced cost savings. However, the intervention still resulted in QALY gains and was cost saving compared to no intervention (Table 5). The analysis of booster sessions indicated that the impact of extending the intervention effects over all years was large on QALYs gained and societal costs, for both interventions: approximately 2.3 times as large in terms of QALY gains and 2.5 times as large in terms of cost savings.

In all analyses, both interventions resulted in more QALYs gained and lower societal costs compared to no intervention. The interventions were cost saving independent of time perspective and thus provide a cost-efficient use of resources compared to no intervention. When comparing senior meetings with a preventive home visit, the senior meetings resulted in both more QALYs gained and lower societal costs indicating that senior meetings was the most cost-effective intervention. In terms of days in full health, the QALYs gained for senior meetings amounted to 20 days (main analysis), 15 days (sensitivity analysis, reduced intervention effect) or 45 days (booster sessions).

## Discussion

The results of our study show that health promotion for community-dwelling older people is cost-effective both in the format of senior meetings and in the format of a preventive home visit. The size of intervention effect on QALYs gained could be considered low based on the follow-up period of 4 years, but is consistent with findings from a review of cost-utility analysis in which the median QALY gain was 0.06 (Wisløff et al. 2014). When extrapolating the effect to hypothetical cohorts of 100 people, the interventions resulted in approximately 5 QALYs gained for both interventions compared to no intervention and approximately 230,000 € and 210,000 € in reduced societal costs for senior meetings and a preventive home visit, respectively. Thus, by implementing senior meetings and/or a preventive home visit, societal costs can be reduced allowing resources to be used for the implementation of other health and/or social care interventions.

When the two interventions are each compared to no intervention, the senior meetings resulted in both larger QALY gains and lower societal costs than the preventive home visit and as such, senior meetings would be the primary choice for implementation. However, in terms of implementation, it has been found that a majority of potential participants are likely to decline to participate due to a lack of interest, lack of time or due to poor health (Gustafsson et al. 2012; Zingmark et al. 2014). Qualitative studies (Behm et al. 2013a, b) have shown that the decision to participate in an intervention was based on the fact that either potential participants experienced the intervention to be too “demanding” because they had symptoms that hindered them, or they considered themselves to be too healthy and accordingly not a target group for the intervention. Therefore, it will be important to consider how health promotion can be tailored in a way that evokes interest among potential participants, for example, by offering the possibility to choose which type of intervention the person prefers to participate in (Dapp et al. 2011) or minimizing the number of sessions (Zingmark et al. 2016a). Thus, rather than identifying the (one) most effective and cost-effective intervention, a more relevant goal could be to identify a “smorgasbord”, i.e. a range of effective interventions so as to reach a high proportion of potential participants. However, the issue of clearly defining the target population and identifying effective interventions specifically tailored for that group remains. In addition, in order to reach suitable target groups for which health promotion can be effective, feasible methods for screening, including self-report, should be considered (Dapp et al. 2014; Dahlin-Ivanoff et al. 2016).

Given that senior meetings, a preventive home visit and booster sessions all were cost-effective, these interventions could be considered for implementation. However, preventive home visits is an intervention that has been studied over 3 decades, yielding conflicting results whether the intervention is effective or not, or of it at all should be recommended for implementation (Mayo-Wilson et al. 2014). However, in the trial on which the intervention effect for this study was based, both senior meetings and a preventive home visit had a person-centred approach and the participants described, as active components, the focus on every person’s needs and how it strengthened the participants self-esteem and self-efficacy (Behm et al. 2013a, b). In addition, in the senior meetings, the group model, including peer-learning, was a way of empowering the participants giving them role models and a sense of sharing problems with persons in similar circumstances. Moreover, the multi-professional approach, where persons from different professions work together, mediates a broad spectrum of information. In conclusion, both interventions had the same theoretical approach, but the differences in format and core components could explain why senior meetings resulted in more QALYs and societal costs gained in comparison with preventive home visit.

The results indicated that booster sessions resulted in additional effects for both senior meetings and a preventive home visit. The results presented in Table 6 indicate that from the second year to the fourth year, a larger proportion in both intervention groups transition from mild to moderate dependency than in the control group, a result further indicating that booster sessions should be recommended. Previously, Sahlen et al. (2006) demonstrated that the effects were sustained only for as long as preventive home visits were implemented, in their trial over 2 years. Taken together, also supported by others demonstrating positive effects of booster sessions (de Boer et al. 2014; Fu et al. 2015) this is an incentive for the implementation of booster sessions in health promotion practices.

When booster sessions were included in the analysis, only those who remained in the mild dependency state participated in the subsequent intervention. This hypothetical construct could be considered logical from a clinical perspective since health promotion optimally targets pre-frail older people without major functional limitations (Dahlin-Ivanoff et al. 2010). However, also for those experiencing a decline it is important to consider which types of intervention could be appropriate in terms of health promotion of secondary prevention. For example, for persons who are at risk of becoming dependent in ADL, it has been shown that reablement is effective in terms of improved ADL ability, physical function and a reduced need for home care (Tessier et al. 2016).

### Methodological considerations

The results need to be interpreted in relation to the modelling approach used, i.e. the validity of the results is dependent on the fit between the model and the real world (Pouryamout et al. 2012). The model does not include a “no dependency state” which could be considered a limitation. However, in the Canadian classification system for disability, on which transition probabilities were calculated, the mildest profile (equal to our state mild dependency) was defined by difficulties in IADL (e.g. housekeeping and occasional heavy jobs such as painting and shovelling snow). Therefore, and considering the population under study, older people at risk for functional decline, mild dependency was chosen as the least dependent state. Nevertheless, it should be noted that any model, including ours, is a simplification of the real world not fully acknowledging individual situations.

One critical parameter in our model is the assumption concerning transition probabilities between states of dependency (Raîche et al. 2012). Based on empirical data from a Swedish randomized controlled trial (Dahlin-Ivanoff et al. 2010), the present study is the first to validate the transition probabilities for the first year upon which the model is built. The probabilities for transitions after 1 year follow a similar pattern in the control group in the study by Dahlin-Ivanoff et al., as in the cohort study by Raîche et al. Based on data from Table 3, the probability for stability in mild dependency in the control group after 1 year is 0.76 (87/114) which is similar to the transition probabilities presented in Table 1 (based on Raiche et al.). However, we acknowledge that the transition probabilities are not exactly same in the Canadian and Swedish cohorts and additional longitudinal cohort studies could further enhance the precision of the model.

Another critical parameter is the intervention effect. As shown in a previous study (Zingmark et al. 2016b), rather than the intervention cost, the most important factor for the magnitude of QALY gains and cost savings was the intervention effect. The estimates of intervention effects used in this trial are based on a randomized controlled trial in which the results are well in line with those of previous health promoting interventions (Sahlen et al. 2006; Beswick et al. 2008; Zingmark et al. 2014). Therefore, we consider our estimates of intervention effects to be reliable. In relation to the other societal costs involved, i.e. informal care, health care, social care, accommodation (Lindholm et al. 2013), the cost for a short-term health promoting intervention is relatively low. While recent trials indicate that a short-term health promoting intervention is sufficient to yield positive effects on a range of outcomes (Gustafsson et al. 2012; Zidén et al. 2013; Behm et al. 2014; Zingmark et al. 2014), it is clearly relevant to further explore how different forms of health promoting interventions should be optimally designed to be feasible to implement and to maintain intervention effects over time. Additional limitations concern the estimates of HRQoL scores, more specifically (1) that these estimates were based on different instrument (SF-12 and EQ-5D) and (2) that the estimates of HRQoL for moderate, total and severe dependency were based on studies referring to decrements in HRQoL due to loss of function and independence rather than estimates for each specific health state.

## Conclusion

This study demonstrates that health promotion implemented for community-dwelling older people in the format of senior meetings or a preventive home visit is cost-effective. Both interventions lead to QALY gains and reduce societal costs at any follow-up over 4 years, and thus, resources can be used to implement other interventions. In contrast to other societal cost such as health and social care, the cost for any of the two studied interventions is very small. Rather than the intervention cost, the most important factor for the magnitude of QALY gains and cost savings was the intervention effect. In addition, yearly booster sessions implemented for those persons who have maintained their level of functioning extend the intervention effects adding additional QALYs and further reducing societal costs.
